# Genome Sequencing and Assembly of Enterotoxigenic *Escherichia coli* E9034A: Role of LngA, CstH, and FliC in Intestinal Cell Colonization and the Release of the Proinflammatory Cytokine IL-8

**DOI:** 10.3390/microorganisms13020374

**Published:** 2025-02-08

**Authors:** Ricardo Rodríguez-Martínez, Sara A. Ochoa, Ricardo Valle-Rios, Gustavo A. Jaimes-Ortega, Rigoberto Hernández-Castro, Jetsi Mancilla-Rojano, Graciela Castro-Escarpulli, Catalina López-Saucedo, Teresa Estrada-García, Ariadnna Cruz-Córdova, Juan Xicohtencatl-Cortes

**Affiliations:** 1 Departamento de Microbiología, Escuela Nacional de Ciencias Biológicas, Instituto Politécnico Nacional, Mexico City 11340, Mexico; www.birec@gmail.com (R.R.-M.); chelacastro@hotmail.com (G.C.-E.); 2 Laboratorio de Investigación en Bacteriología Intestinal, Hospital Infantil de México Federico Gómez, Mexico City 06720, Mexico; saraariadnah@hotmail.com (S.A.O.); mancillajetsi@gmail.com (J.M.-R.); 3 Unidad Universitaria de Investigación en Cáncer e Inmunología, División de Investigación, Facultad de Medicina, Universidad Nacional Autónoma de México, Mexico City 04510, Mexico; vallerios@unam.mx (R.V.-R.); gelet321@gmail.com (G.A.J.-O.); 4 Unidad de Investigación en Inmunología y Proteómica, Hospital Infantil de México Federico Gómez, Mexico City 06720, Mexico; 5 Posgrado en Biología Experimental, Departamento de Ciencias Biológicas y de la Salud, Universidad Autónoma Metropolitana, Mexico City 09310, Mexico; 6 Departamento de Ecología de Agentes Patógenos, Hospital General Dr. Manuel Gea González, Mexico City 14080, Mexico; rigo37@gmail.com; 7 Posgrado en Ciencias Biológicas, Facultad de Medicina, Universidad Nacional Autónoma de México, Mexico City 04510, Mexico; 8 Departamento de Biomedicina Molecular, Centro de Investigación y de Estudios Avanzados CINVESTAV-IPN, Mexico City 07360, Mexico; calopez@cinvestav.mx (C.L.-S.); testrada@cinvestav.mx (T.E.-G.); 9 Laboratorio de Investigación en Inmunoquímica, Hospital Infantil de México Federico Gómez, Mexico City 06720, Mexico

**Keywords:** enterotoxigenic *Escherichia coli*, colonization factor, cytokine, toxin

## Abstract

Enterotoxigenic *Escherichia coli* (ETEC) produces two types of enterotoxins, LTs and STs, as well as several colonization factors (CFs), including CS21, CS3 fimbriae, and flagellar structures. This study investigated how these structures contribute to ETEC colonization and the immune response in HT-29 and HuTu-80 intestinal cells. ETEC strains with single, double, and triple mutations in the *lngA*, *cstH*, and *fliC* genes were generated and confirmed using PCR and Western blotting. The colonization of HT-29 and HuTu-80 intestinal cells by the ETEC E9034A strain, which was fully sequenced using a hybrid approach involving both Illumina and Oxford Nanopore technologies, was used to generate the mutant and recombinant proteins. The colonization and adherence of E9034A and its mutants were assessed through colony-forming unit (CFU) counts. Cytokine levels were assessed using flow cytometry and analyzed via FlowJo 7.6.1. Quantitative analysis revealed that the absence of the *lngA*, *cstH*, and *fliC* genes significantly (*p* < 0.01) reduced ETEC adherence to HT-29 and HutU-80 cells. In addition, only ETEC strains expressing the FliC protein induced IL-8 secretion. These findings suggest that LngA, CstH, and FliC in ETEC E9034A enhance adherence to intestinal cells and trigger the release of IL-8.

## 1. Introduction

Diarrheal diseases cause approximately 446,000 deaths annually, primarily affecting children under five years old, newborns, and older adults in low-income countries [1,2]. Enterotoxigenic *Escherichia coli* (ETEC) is one of the most important pathogens, and it has been estimated that it causes an average of 220 million episodes of diarrhea globally. Despite global development, several countries still lack or do not have access to purified drinking water or sanitation. In these countries, the morbidity and mortality rates due to ETEC continue to be important health problems and represent an essential challenge for pediatric health [3,4,5,6].

In Mexico, diarrheal diseases are the second most common cause of morbidity in children from 0 to 5 years of age [7]. Although the mortality rate has decreased since the introduction of the rotavirus vaccine, the morbidity rate still represents a significant health challenge associated with unfavorable living conditions [7,8]. Fecal contamination of water and food is considered the primary source of ETEC infection, and the human infectious dose for ETEC is estimated to be 10^8^ colony-forming units (CFUs) [9,10]. The virulence of ETEC is driven primarily by the production of two types of enterotoxins, heat-labile toxins (LTs) and heat-stable toxins (STs), which cause electrolyte imbalances and damage to epithelial cells in the small intestine [9,11,12]. Toxins released by the bacterium are secreted into the intestinal epithelium upon infection, leading to watery diarrhea [13]. STs have two variants, STa and STb, while LTs consist of a catalytic A subunit and five B subunits, which are the primary causes of diarrhea symptoms [14,15]. Several colonization factors (CFs) play a significant role in the virulence of ETEC and could be potential antigens for the development of preventive vaccines against this intestinal pathogen. ETEC strain E9034A belongs to the ETEC lineage and is widely distributed in various geographical areas and time periods. These ETEC strains exhibit significant genetic and phenotypic variation in their CFs, allowing them to adapt effectively to both animal and human hosts. Genomic studies suggest that the expression of one, two, or three CFs consistently correlates across several conserved operons in ETEC strains, which share genetic material associated with similar or combined toxin and colonization factor profiles. This adaptability may be linked to specific regions where coexpressed CFs, such as CS21 and CS3, along with the flagellum and occasionally CS6, are essential for colonization in humans [16,17]. The CFs of ETEC, including CFA/I, CFA/II, and CFA/IV, are fimbrial structures that allow the bacterium to adhere to the intestinal epithelium in the host. This adherence facilitates colonization and leads to infection. Recent studies have revealed a close relationship between the CFs identified in ETEC strains isolated from humans and those from animals. These data indicate that horizontal transfer mechanisms enable the exchange of genetic information between strains, promoting infection across various hosts and enhancing the adaptability and persistence of ETEC in diverse environments [12,18]. Gaining insights into these CFs will allow us to trace the evolution of these pathogens and create effective strategies for preventing and controlling intestinal infections, including the development of effective vaccines against diarrheal diseases [19,20,21]. ETEC assembles a type IV pilus known as CS21, or longus, which is located at one pole of the bacterial cell. The CS21 pilus has a diameter of 7 nm and can extend over 20 µm in length. This filament is composed of the LngA protein, which has a molecular weight of 22 kDa and is encoded by the *lngA* gene located in a 14 kb megaplasmid called *lng*. Previous studies have shown that the LngA protein of the CS21 pilus is a key virulence factor in clinical ETEC strains across various regions worldwide [17,21,22]. Our group demonstrated that the CS21 pilus mediates the colonization of intestinal cells and that bacterial aggregation is stable in intimate interactions with intestinal epithelial cells [23,24]. The high frequency of CS21 in ETEC strains suggests that this polymeric molecule significantly participates in the immune response; however, further study is needed to elucidate its function [25].

The CS3 fimbriae of ETEC are small and flexible structures that measure approximately 2 nm in diameter. These fimbriae are composed mainly of the 14-kDa CstH protein, which plays a crucial role in adherence to intestinal epithelial cells and immune response modulation [26,27,28]. The ETEC flagellar machinery is a structure comprising several proteins that are involved primarily in motility, and the conserved region consists primarily of flagellin (FliC), which has a molecular weight of 69 kDa. Several studies have indicated that EtpA, an adhesion protein, assembles in the distal part of the flagellum of ETEC. This protein plays a critical role in epithelial cell colonization, facilitating close interactions with enterocytes and triggering an immune response [29,30,31]. Studies using human cell lines, such as HT-29 and HuTu 80, will improve the understanding of how ETEC modulates the host immune response and activates different signaling pathways during intestinal disease [32]. Currently, there are no licensed vaccines for ETEC-induced diarrhea [21,25,31]. This study aimed to elucidate how CS21, CS3, and flagella from ETEC contribute to colonization and their potential role in cytokine release in HT-29 and HuTu 80 intestinal cells. This information will allow us to identify potential therapeutic targets to combat this pathogen and reduce its global impact on pediatric health.

## 2. Materials and Methods

### 2.1. Bacterial Strains

The ETEC bacterial strains (wild-type and mutant) used in this study are described in Table 1. The ETEC E9034A and mutant (with nonpolar deletion of the *lngA*, *fliC*, and *cstH* genes) strains were grown on Luria Bertani (LB) agar or broth (BD; New Jersey, USA) supplemented with kanamycin (50 µg/mL) or/and chloramphenicol (50 µg/mL) as needed with overnight incubation at 37 °C. The strains were stored in LB broth supplemented with glycerol (20%) at −70 °C until use.

### 2.2. DNA Extraction and Genomic Data Processing

Genomic DNA from the ETEC strain E9034A was extracted with the phenol—chloroform method. Whole-genome sequencing for the strain was carried out following the standard Illumina TruSeq protocol, employing paired-end reads (2 × 75 bp) with the Illumina NextSeq500 platform, complemented with sequencing reads from the Oxford Nanopore MiniON platform. Hybrid assembly was performed using Unicycler v0.4.1 [33], and functional annotation of the sequences was carried out via Prokka v1.2.0 in parallel with the RAST platform (https://rast.nmpdr.org/rast.cgi, accessed on 3 October 2024) [34,35]. Finally, the generated data were deposited in the NCBI database.

The genomic sequences were analyzed and visualized using the Proksee platform (https://proksee.ca, accessed on 3 October 2024), and a BAKTA tool (https://bakta.computational.bio, accessed on 3 October 2024) was used for coding sequence annotation [36]. Genome diagrams for the central chromosome and the three circularized plasmids were constructed, and the identification of coding genes and key genomic regions was performed, with Proksee (https://proksee.ca, accessed on 3 October 2024). Horizontally transferred genes were identified using Alien Hunter (https://www.sanger.ac.uk/tool/alien_hunter/, accessed on 3 October 2024), which detects genomic regions potentially acquired through horizontal transfer and associates regions with replication origins such as oriC. GC content and CG skew analysis were performed with Proksee’s integrated GC Skew tool (https://proksee.ca, accessed on 3 October 2024), enabling the identification of replication origins and termination sites, together with regions with notable nucleotide content variation. Antibiotic resistance genes were identified through the CARD v1.2.1 (Comprehensive Antibiotic Resistance Database, https://card.mcmaster.ca, accessed on 3 October 2024). Prophage sequences were analyzed with the Phigaro tool v2.3.0, which identifies and maps prophage regions within the bacterial genome. CRISPR—Cas system identification was carried out using the program CRISPRCasFinder (https://crisprcas.i2bc.paris-saclay.fr/CrisprCasFinder/Index, accessed on 3 October 2024). The *lngA*, *cstH* and *fliC* genes were identified, mapped, and used to design primers for amplification, mutation, and cloning for recombinant protein expression.

### 2.3. Construction of an Isogenic ETEC Mutant

The *lngA*, *cstH*, and *fliC* genes were deleted from E9034A via the Lambda—Red recombination method described by Datsenko and Wanner (2000) [37]. The flanking primers used for deletion of the *lngA*, *cstH*, and *fliC* genes are described in Table 2. Gene deletion was confirmed by a polymerase chain reaction (PCR) with Platinum^®^ Taq DNA Polymerase, High Fidelity^®^, enzyme mix (Thermo Fisher Scientific; Carlsbad, CA, USA).

### 2.4. Antibody Production and Western Blotting

Expression of the LngA, CstH, and FliC proteins in the wild-type ETEC strain E9034A and the ETEC strains with mutations in the *lngA*, *cstH*, and *fliC* genes was detected by Western blotting. The primers used for the generation of constructs for the expression of the recombinant proteins LngA, CstH, and FliC are described in Table 2. The *lngA*, *cstH*, and *fliC* genes were cloned and inserted into the pLATE31 plasmid according to the aLICator Ligation Independent Cloning and Expression System (Thermo Scientific, Santa Clara, CA, USA). The plasmid pLATE31 and the recombinant plasmids were transformed into *E. coli* BL21(DE3). Protein expression was induced with 1 mM isopropyl 1-thio-ß-galactopyranoside (IPTG), followed by growth for 5 h at 37 °C. The recombinant proteins labeled with His-Tag were purified as previously described by Saldaña-Ahuactzi, 2016 [22].

Anti-CS21, anti-CS3, and anti-FliC sera were produced by immunizing New Zealand rabbits with the purified recombinant proteins LngA, CstH, and FliC labeled with a C-terminal His-Tag. The rabbits were subcutaneously immunized every two weeks. The protocol included one dose of 1 µg of antigen emulsified in 500 µL of Freund’s complete adjuvant, followed by three doses of 0.5 µg of antigen in 500 µL of PBS emulsified in Freund’s incomplete adjuvant.

The obtained sera were used for Western blotting assays. Briefly, bacterial strains were grown in 5 mL of LB medium overnight with shaking at 37 °C. The bacterial cultures were adjusted to an optical density (OD_600_) of 1.0 and centrifuged at 4000× *g* for 5 min. The obtained bacterial pellets were resuspended in 200 µL of loading buffer and lysed at 95 °C for 10 min. The protein extracts were subjected to 16% sodium dodecyl sulfate—polyacrylamide gel electrophoresis (SDS—PAGE). Proteins separated by electrophoresis were subsequently transferred to nitrocellulose membranes, which were blocked with PBS supplemented with 0.1% Tween 20 (*v*/*v*) and 5% nonfat dry milk. The membranes were incubated for 1 h with polyclonal rabbit antibodies (anti-CS21, anti-CstH, and anti-FliC) diluted 1:10,000 in PBS-0.1% Tween, washed three times with PBS-0.1% Tween, and incubated for 1 h with peroxidase-conjugated goat anti-rabbit IgG (Sigma—Aldrich-Co., St. Louis, MO, USA) diluted 1:10,000 in PBS-0.1% Tween. The membranes were washed again three times with 1× PBS-Tween and visualized using an Immobilon Western Chemiluminescent HRP Substrate (Amersham Life and Science; Arlington Heights, IL, USA) [22].

### 2.5. Adherence Assays

Intestinal HT-29 cells, a human colorectal adenocarcinoma cell line (ATCC HTB-38) derived from a primary tumor, and HuTu 80 cells, a human duodenal adenocarcinoma cell line (ATCC HTB40) (ATCC^®^; Manassas, VA, USA) derived from a duodenal tumor, were used in this study. The intestinal HT-29 cells were seeded in 75 cm^2^ cell culture flasks with high-glucose Dulbecco’s modified Eagle’s medium (DMEM, ATCC; Manassas, VA, USA), and HuTu 80 cells were seeded with Eagle’s minimal essential medium (EMEM, ATCC; Manassas, VA, USA), and grown until they reached 80% confluence. Then, 1 × 10^5^ cells/mL were seeded into each well of 24-well polystyrene plates (Corning, NY, USA) and incubated at 37 °C under 5% CO_2_. The cells were subsequently infected with the bacterial strains at a multiplicity of infection (MOI) of 1:100 and incubated for 4 h at 37 °C under 5% CO_2_ [24].

After infection, the supernatants were removed, the monolayers containing the bacteria were washed with 1× PBS and treated with 0.1% Triton X-100 (Amresco; Solon, OH, USA), and serial dilutions were quantitatively analyzed. The dilutions were spotted on LB agar to determine the CFU/mL ratio. The assays were performed in triplicate on three different days.

### 2.6. Quantification of Pro- and Anti-Inflammatory Cytokines via Flow Cytometry

The HT-29 and HuTu 80 cell lines were infected with the ETEC strain E9034A, the mutant strains, or *E. coli* BL21 at an MOI of 1:100 and incubated at 37 °C under 5% CO_2_ for 4 h. After incubation, the supernatants were recovered and processed according to the instructions of the BD™ Cytometric Bead Array (CBA) Human Inflammatory Cytokine Kit (BD Biosciences, San Jose, CA, USA). The assays were performed in triplicate on three different days to reduce experimental errors and ensure reproducibility. The kit’s capture and detection reagents were prepared for different assays and included different microspheres specific for the cytokines IL-8, IL-1β, IL-6, IL-10, TNF, and IL-12p70. The samples were incubated with the prepared reagents according to the duration and temperature guidelines recommended in the manual to ensure specific interactions between the cytokines and microspheres. Data acquisition was performed via a BD Biosciences FACS Canto II flow cytometer (BD Biosciences; San Jose, CA, USA) to detect the microspheres and specifically quantify the cytokine levels, following the manufacturer’s guidelines.

### 2.7. Statistical Analysis

The data generated from the quantitative analysis in the adherence and flow cytometry assays are expressed as the means and standard errors of the means (SEMs). The statistical analyses were conducted via R software (v 4.2.3) [38]. Comparisons between groups in the adherence assays were assessed via two-way ANOVA. For flow cytometry data, a nonparametric Kruskal—Wallis test was used. In both cases, a *p* value < 0.05 was considered to indicate statistical significance.

## 3. Results

### 3.1. Genomic Information of the ETEC E9034A Strain

A total of 4,620,542 reads were generated using the Illumina NextSeq500 platform, with paired-end reads measuring 2 × 75 bp. Additionally, Oxford Nanopore sequencing with the MinION platform produced 86,098 reads, with an average read length of 1374 bp and an N50 of 3032 bp, resulting in a total of 118,303,228 bases sequenced. The reads from both platforms were subjected to hybrid assembly via Unicycler. The Illumina reads were processed in paired-end format, whereas the long reads from the Oxford Nanopore platform were processed in single-end format. The average coverage for both long and short reads was 40×. The longest contig was 4,801,745 bp, with an N50 of 4,801,745 bp. The assembly successfully closed the chromosome and included three additional contigs corresponding to plasmids. The GC content was 50.53%, which is characteristic of *E*. *coli*.

**Figure 1:**
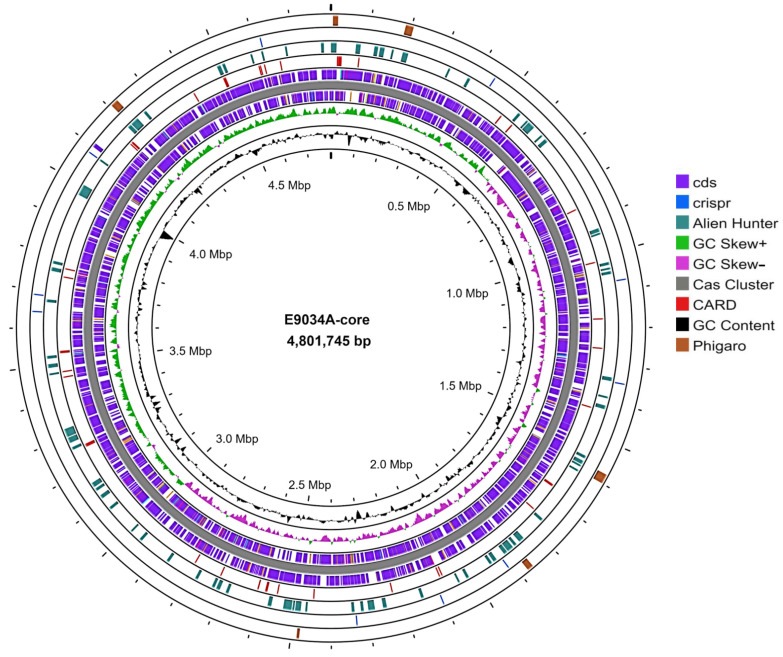
Visualization of the core genome of the ETEC strain E9034A. The PROKSEE platform was used for visualization of the core genome. Annotation was conducted to identify coding sequences (CDSs), which are represented in purple. Horizontally transferred gene sequences were identified using Alien Hunter and are highlighted in bright green. A complete CRISPR—Cas system was established and is shown in blue. The GC content was calculated with the GC skew, displayed in green and purple, whereas the overall GC content is represented in black. Six bacteriophages, indicated in brown, were detected using the Phigaro tool v1.0.1., and antibiotic resistance-associated sequences, highlighted in red, were identified using CARD.

**Figure 2:**
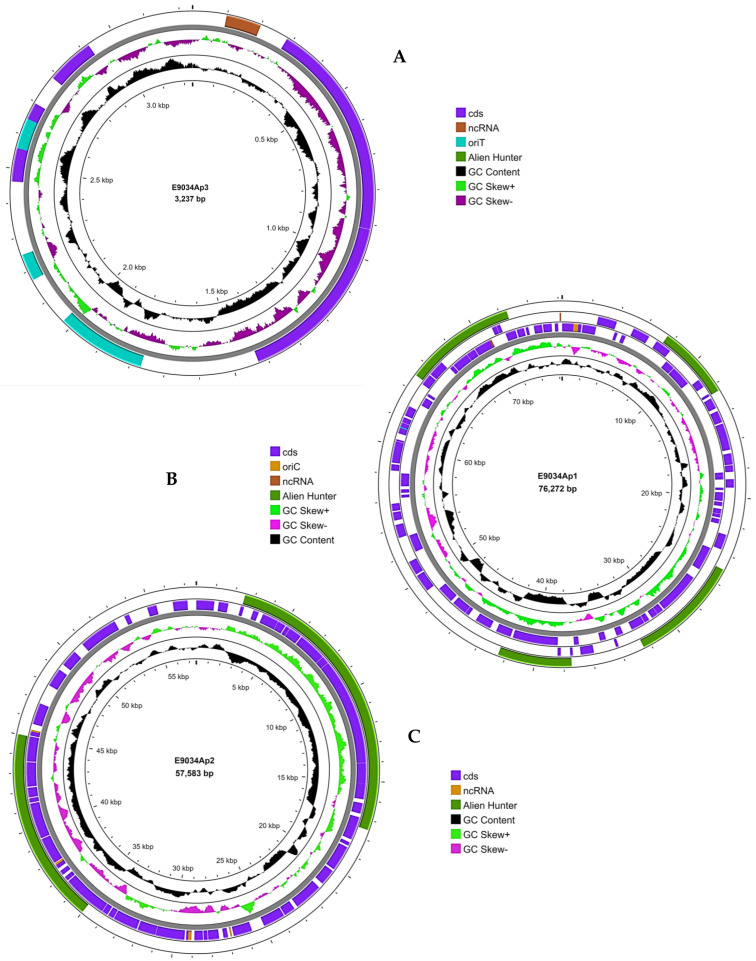
Visualization of plasmids from the ETEC strain E9034A. Three plasmids were identified in the ETEC E9034A strain: (**A**) 3237 bp, (**B**) 76,273 bp, and (**C**) 57,583 bp. The PROKSEE platform was used to visualize the plasmids. Annotation was performed to identify coding sequences (CDSs), which are represented in purple. Horizontally transferred gene sequences were identified using Alien Hunter and are highlighted in bright green. The GC content was calculated with the GC skew, shown in green and purple, whereas the overall GC content is displayed in black. Six bacteriophages, indicated in brown, were detected using the Phigaro tool, and antibiotic resistance-associated sequences, highlighted in red, were identified through CARD.

The complete genome of the ETEC strain E9034A was deposited in the NCBI database, including its chromosome (accession: CP171231), with a length of 4,801,745 bp, and three plasmids (accession: CP171232, CP171233, and CP171234), with sizes of 76,272 bp, 57,583 bp, and 3237 bp, respectively. This genomic information is associated with BioProject PRJNA1171086. Genome annotation using the RAST platform (https://rast.nmpdr.org/, accessed on 3 October 2024) revealed 4779 coding sequences, 108 RNAs, and 599 subsystems, highlighting categories associated with carbohydrates, cofactors, vitamins and pigments, amino acids, and defense. CARD tool analysis on the Proksee platform identified at least 53 sequences associated with antibiotic resistance genes. The Alien Hunter tool revealed 76 sequences in the core genome linked to horizontal gene transfer, and at least one sequence in the plasmid exhibited an independent replication origin, such as oriC, oriT, or ncRNA. The Phigaro tool identified six sequences related to bacteriophages that were inserted into the core genome. Analysis using CRISPRCasFinder revealed that the core genome contained a CAS-type IE system, which was not found in the plasmids. The core genome is illustrated in Figure 1, while the plasmids are depicted in Figure 2.

The *lngA* and *cstH* genes were identified in a megaplasmid (CP171233), and the *fliC* gene was identified in the chromosome. The *lngA* gene encodes the LngA structural subunit of CS21 fimbria, is located in a megaplasmid, spans positions 5361 to 6071, and has a length of 711 bp. The *cstH* gene encodes the structural subunit of the CS3 fimbria, is located in a megaplasmid between positions 30,450 and 30,956, and covers 507 bp. The *fliC* gene encodes the FliC structural subunit of the flagellum, is located in the chromosome between positions 176,566 and 178,578, and has a length of 2013 bp. The *lngA*, *cstH*, and *fliC* gene sequences were used to design primers for generating mutant and recombinant proteins for expression.

### 3.2. Identification of the LngA, CstH, and FliC Proteins in ETEC E9034A and Its Derived Mutants

**Figure 3:**
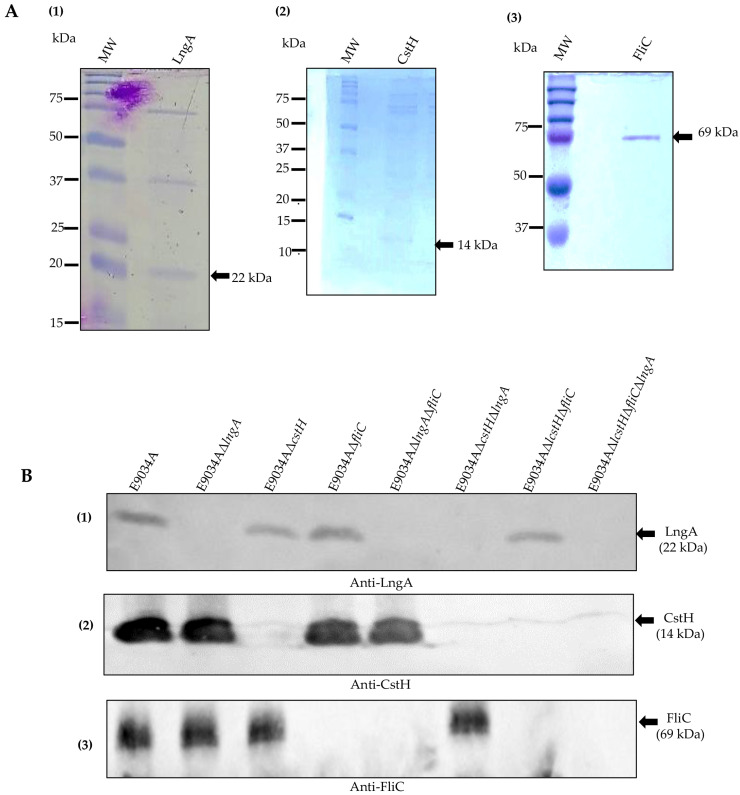
Identification of the LngA, CstH, and FliC proteins by SDS-PAGE gels and Western blotting. (**A**) Coomassie blue-stained gels: (**1**) Purified LngA protein of 22 kDa. (**2**) Purified CstH protein of 14 kDa. (**3**) Purified FliC protein of 69 kDa. (**B**) Immunodetection of the LngA, CstH, and FliC proteins: (**1**) Strains E9034A, E9034AΔ*cstH*, E9034AΔ*fliC*, and E9034AΔ*cstH*Δ*fliC* expressed a 22 kDa protein corresponding to the CS21 pilus. (**2**). Strains E9034A, E9034AΔ*lngA*, E9034AΔ*fliC*, and E9034AΔ*lngA*Δ*fliC* expressed a 14 kDa protein corresponding to the CS3 fibrils. (**3**) Strains E9034A, E9034AΔ*lngA*, E9034Aδ*cstH*, and E9034AΔ*cstH*Δ*lngA* expressed the FliC protein of 69 kDa.

Mutations in the *lngA*, *cstH*, and *fliC* genes were evaluated by PCR to confirm the deletion of these genes. The integrity and purity of the proteins were assessed for the ETEC strain E9034A and its mutants through 16% SDS—PAGE and Western blotting (Figure 3). To increase the specificity of the antibodies, polyclonal rabbit sera produced from the purified proteins (LngA, CstH, and FliC) were adsorbed sevenfold using the respective mutant strains, i.e., the serum generated from the LngA protein was absorbed by the *lngA* mutant strain.

Analysis of 16% SDS—PAGE gels stained with Coomassie blue revealed that the LngA protein had a molecular weight of 22 kDa (Figure 3A(1)), the CstH protein had a molecular weight of 14 kDa (Figure 3A(2)), and the FliC protein had a molecular weight of 69 kDa (Figure 3A(3)). Western blot analysis revealed a 22 kDa protein, corresponding to the LngA protein in the E9034A, E9034AΔ*cstH*, E9034AΔ*fliC*, and E9034AΔ*cstH*Δ*fliC* strains. The knockout strains E9034AΔ*lngA*, E9034AΔ*lngA*Δ*fliC*, and E9034AΔ*lngA* Δ*cstH*Δ*fliC* did not express the LngA protein (Figure 3B(1)). The ETEC strains E9034A, E9034AΔ*lngA*, E9034AΔ*fliC*, and E9034AΔ*lngA*Δ*fliC* expressed a 14 kDa protein corresponding to the *CstH* protein; however, this protein was not identified in the knockout strains E9034AΔ*cstH*, E9034AΔ*cstH*Δ*lngA*, E9034AΔ*cstH*Δ*fliC*, and E9034AΔ*lngA*Δ*cstH*Δ*fliC* (Figure 3B(2)). The 69 kDa FliC protein was identified in a set of ETEC strains (E9034AΔ*lngA*, E9034Aδ*cstH*, and E9034AΔ*lngA*Δ*cstH*) in which the *fliC* gene was not deleted. In contrast, ETEC strains in which the *fliC* gene was replaced with a kanamycin cassette did not express this protein (Figure 3B(3)).

### 3.3. Inactivation of the lngA, cstH, and fliC Genes in ETEC E9034A Significantly Reduces Adherence to HT-29 Cells

**Figure 4:**
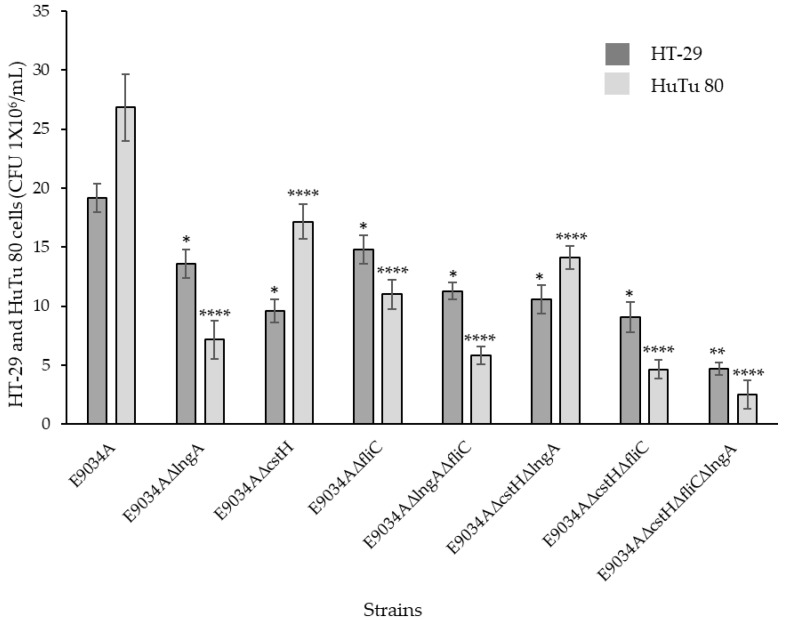
Quantitative analysis of the adherence of ETEC strains to HT-29 and HuTu 80 cells. The absence of the genes *lngA*, *cstH*, and *fliC* affected bacterial adherence to HT-29 and HuTu 80 cell monolayers. Compared with the single mutants, the double mutants did not show significant changes in adherence percentages. Compared with the E9034A and double-mutant strains, the ETEC triple-mutant strain exhibited a significant reduction in adherence on both cellular lines. The graph depicts the final values derived from the adherence data in the HT-29 and HuTu 80 cells, subtracting the data obtained without cells for each bacterial strain. The mean ± SEM values are presented; (* *p* < 0.05 and ** *p* < 0.001 for HT-29 cells) and (**** *p* < 0.0001 for HuTu 80 cell), both compared with cells infected with the E9034A strain.

Compared with those of E9034AΔ*cstH*Δ*fliC*Δ*lngA* and E9034AΔ*lngA*, the adherence of the ETEC strains E9034A, E9034AΔ*cstH*, E9034AΔ*lngH*, and E9034AΔ*lngA* was reduced by 50% to 40% (Figure 4). The E9034AΔ*fliC*Δ*cstH*, E9034AΔ*cstH*Δ*lngA*, and E9034AΔ*fliC*Δ*lngA*, E9034AΔ*lngA*, and E9034AΔ*fliC* strains presented reductions in adherence ranging from 30% to 23% (Figure 4). Additionally, the ETEC strain E9034AΔ*cstH* exhibited a 20% reduction in adherence compared with the ETEC strain E9034AΔ*lngA*. Nevertheless, no differences were detected in the percentages of adherence to HT-29 cells between the ETECE9034AΔ*cstH* strain and the E9034AΔ*lngA*Δ*fliC*, E9034AΔ*lngA*Δ*cstH* and E9034AΔ*cstH*Δ*fliC* strains (Figure 4). The adherence of the E9034AΔ*lngA*Δ*cstH*Δ*fliC* strain to HT-29 intestinal cells decreased by an average of 50% to 30% compared with that of the single-mutant strains and by 52% to 47% compared with that of the double-mutant strains (Figure 4). Furthermore, the triple mutant displayed a 79% reduction in adherence to HT-29 cells (Figure 4). The numerical values of the adherence assay tests are shown in Supplementary Material File S1.

### 3.4. Role of the LngA, CstH, and FliC Proteins in ETEC Strains During the Adherence of HuTu 80 Cells

Compared with that of the ETEC strain E9034A, the adherence percentage of the ETEC E9034AΔ*lngA* strain to HuTu 80 cells decreased by 73%, that of the E9034AΔ*cstH* strain decreased by 36%, and that of the E9034AΔ*fliC* strain decreased by 59%. Significant reductions in adherence percentage were observed for the strains E9034AΔ*cstH*Δ*fliC* (82%), E9034AΔ*lngA*Δ*fliC* (78%), and E9034AΔ*lngA*Δ*cstH* (47%) compared with the ETEC strain E9034A. Compared with the ETEC strain E9034A, the strain E9034AΔ*lngA*Δ*cstH*Δ*fliC* exhibited a 90% reduction in adherence to HuTu 80 cells. Quantitative analysis revealed that strains with mutations in the *lngA* and *fliC* genes exhibited significantly reduced adherence to HuTu 80 cells (Figure 4). Additionally, the ability of strain E9034AΔ*lngA*Δ*cstH*Δ*fliC* to adhere to HuTu 80 was significantly reduced by 85% (Figure 4). The numerical values of the adherence assay tests are shown in Supplementary Material File S1.

### 3.5. The Protein FliC Induces the Release of the Cytokine IL-8 in HT-29 Intestinal Cells

**Figure 5:**
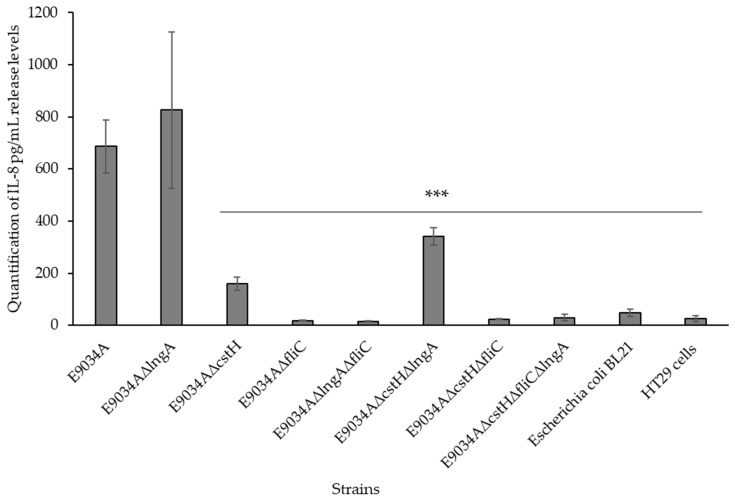
HT29 cells infected with ETEC strains release the cytokine IL-8. In HT-29 cells infected with the ETEC strain E9034A, the concentration of IL-8 produced was approximately 686 pg/mL. Compared with ETEC E9034A, deletion of the *lngA* gene increased the concentration of the cytokine IL-8 to 825 pg/mL. However, the E9034A strain with a mutation in the *cstH* gene significantly reduced the IL-8 concentration to 159 pg/mL. Furthermore, mutation of the *fliC* gene also increased the amount of IL-8 released to 341 pg/mL. The means ± SEMs are presented, with *** *p* < 0.001 compared with cells infected with the ETEC strain E9034A.

To analyze cytokine release, flow cytometry studies were performed using BD™ Cytometric Bead Array (CBA) human inflammatory cytokine kits for IL-8, IL-1β, IL-6, IL-10, TNF, and IL-12p70. The data revealed that the E9034A, E9034AΔ*lngA*, E9034AΔ*cstH*, and E9034AΔ*cstH* strains stimulated only the release of the cytokine IL-8 by HT-29 intestinal cells at concentrations of 686 pg/mL, 825.5 pg/mL, 159 pg/mL, and 341 pg/mL, respectively. Furthermore, the ETEC strain E9034A triggered the release of 4.314-fold more IL-8 than did the E9034AΔ*cstH* strain and 2.011-fold more IL-8 than did the E9034AΔ*cstH*Δ*lngA* strain. Interestingly, compared with strain E9034A, E9034AΔ*lngA* caused a 1.2-fold increase in IL-8 release (Figure 5). In contrast, no IL-8 was detected with the other mutant strains included in this study. A significant reduction (*p* = 0.001) in IL-8 levels was observed when the HT-29 intestinal cells were treated with the E9034AΔ*cstH* strain, resulting in a decrease of 5.188-fold compared with the level observed with the E9034AΔ*cstH*Δ*lngA* strain. In contrast, the E9034AΔ*cstH*Δ*lngA* strain presented a 2.419-fold reduction (*p* = 0.001) compared with the E9034AΔ*cstH*Δ*lngA* strain. In the HuTu 80 cells challenged with the ETEC strains, IL-8 release was not induced after 4 h of incubation.

## 4. Discussion

The fimbrial structures of ETEC provide important insights into the mechanism by which it initially colonizes its host. Understanding the functions of ETEC fimbriae can contribute to the development of new therapeutic targets for treating diarrhea in children and adults [32]. Hybrid sequencing, which combines Illumina and Oxford Nanopore technologies, has proven to be effective for resolving complex bacterial genome sequences [39]. This method allowed us to close the genome of E9034A and identify three plasmids containing key genetic elements associated with virulence and antibiotic resistance.

In this study, we examined the roles of CS21, CS3, and FliC in the ETEC strain E9034A by conducting adherence assays and measuring cytokine activation in HT-29 and HuTu 80 intestinal cells. Single-mutant (E9034AΔ*lngA*, E9034AΔ*cstH*, and E9034AΔ*fliC*), double-mutant (E9034AΔ*lngA*Δ*cstH*, E9034AΔ*lngA*Δ*fliC*, and E9034AΔ*cstH*Δ*fliC*), and triple-mutant (E9034AΔ*lngA*Δ*cstH*Δ*fliC*) strains were generated from the ETEC strain E9034A. Our results reveal that the deletion of *lngA* (E9034Δ*lngA*) and *cstH* (E9034AΔ*cstH*), as well as both genes (E9034AΔ*cstH*Δ*lngA*), affects the capacity of the ETEC strain E9034A to adhere to HT-29 intestinal cells. These data indicate that CS21 and CS3 are two fimbrial structures that directly interact with enterocytes through ligands that have not yet been described. Data generated by our group have indicated that CS21 fimbria are composed primarily of the LngA protein, which plays a role in intestinal cell adherence and bacterial aggregation [22,24,40].

Our group previously proposed that 14 genes or open reading frames within the *lng* operon are involved in the assembly of the CS2 pilus. However, only the functions of the *lngA* and *lngB* genes, which encode the major (LngA) and minor (LngB) structural subunits, respectively, have been defined in detail. Preliminary data suggest that the proteins LngA, LngB, LngC, LngD, LngH, and LngP play crucial roles in the assembly of the CS21 pilus in the ETEC strain E9034A. The absence of these proteins disrupts the polymerization of the CS21 pilus, which impacts the ability of bacteria to colonize HT-29 cells efficiently [22,25]. Additionally, we described how *E. coli* BL21, which carries the *lng* operon with 14 genes, promotes the assembly of the CS21 pilus and enhances its adherence to intestinal cells [41]. Importantly, in vivo models still lack the essential features needed to provide relevant information on the ETEC immune response [8]. Despite global efforts, there are still no effective vaccines available that can reduce ETEC infection [40,42]. The innate immune response plays a primary role in the host’s first line of defense against bacterial pathogens. Toll-like receptors (TLRs), known as pathogen-associated molecular patterns (PAMPs), are the main pattern recognition molecules (PRRs) responsible for detecting conserved microbial components [43]. Other studies have shown that enterocytes in the intestinal epithelium express several types of TLRs, including TLR4 and TLR5. TLRs recognize a wide variety of bacterial components, including lipopolysaccharides (LPSs), lipopeptides, and flagellins; for example, TLR4 recognizes the LPS of Gram-negative bacteria such as *E. coli* [43,44]. These receptors play crucial roles in recognizing the antigenic CFs of bacterial pathogens, such as ETEC, as they enhance the activation of the immune response. Recent studies suggest that ETEC strains that produce both LTs and STs exacerbate epithelial cell damage; however, when ETEC strains produce only STs, a significant decrease in the immune response is observed [45].

LPSs bind to TLR4, interacting with LPS-binding protein (LBP) and CD14 to engage the TLR4/MD-2 complex and triggering the activation of signaling pathways that lead to the production of proinflammatory cytokines, such as IL-6, IL-12, and TNF-α [44,46]. Lipopeptides, lipoproteins, and peptidoglycans produced by *E. coli* are recognized by TLR2 either alone or in association with TLR1 or TLR6 [47,48,49]. Importantly, the activation of TLR2 triggers the production of proinflammatory cytokines. In this study, an infection assay was used to quantify the release of pro- and anti-inflammatory cytokines (IL8, IL1β, IL6, IL10, TNF, and IL12p70) from intestinal cells (HT-29 and HuTu 80) after 4 h of infection with the ETEC strain E9034A and the generated mutants (E9034AΔ*lngA*, E9034AΔ*cstH*, E9034AΔ*fliC*, E9034AΔ*lngA*Δ*fliC*, E9034AΔ*lngA*Δ*cstH*, E9034AΔ*lngA*Δ*fliC*, and E9034AΔ*cstH*Δ*fliC*, and E9034AΔ*lngA*Δ*cstH*Δ*fliC*). Our data indicate that the strains E9034A, E9034AΔ*lngA*, E9034AΔ*cstH*, and E9034AΔ*lngA*Δ*cstH* significantly stimulated the release of IL-8 from intestinal HT-29 cells. Infection with the other mutant strains also significantly reduced the IL-8 level (*p* < 0.001). Other studies have suggested that the ETEC fimbriae CS3 and CS21 modulate IL-8 production, as described in this study [27,28,50]. Flow cytometry analysis of HuTu 80 cells infected with ETEC strains did not detect any release of cytokines. This lack of detection may be due to the timing of the infection or the excess mucus produced by these cells, which could act as a physical barrier. Importantly, there are no previously generated data on HuTu 80 cells infected with ETEC; therefore, further research is needed to determine how the fimbriae and flagella of ETEC strains play essential roles in the release of proinflammatory cytokines, such as IL-6 and IL-8. Under the conditions tested in this study, IL-6 release was not detected in HT-29 or HuTu 80 cells. Nevertheless, the significant increase in IL-8 release from intestinal HT-29 cells may be a key factor that enhances the recruitment of neutrophils to the infection site, exacerbating the inflammatory response. TLR5, located on the basolateral surface of enterocytes, recognizes flagellin, a structural protein of bacterial flagella [51]. This study describes how the ETEC strain with deletion of the *fliC* gene does not induce the release of pro- or anti-inflammatory cytokines. Several studies have shown that the interaction between flagellin and TLR5 activates signaling pathways that lead to the production of IL-8, which recruits neutrophils to the infection site [51,52]. Enterocytes express TLR4 and TLR5, which are recognized by ETEC antigens, such as LPS and flagellin. These factors trigger the activation of signaling pathways that lead to the production of cytokines, such as IL-8, which are essential for the immune response. In this context, our data are consistent with the significant increase in IL-8 observed in flagellin-producing strains, such as E9034AΔ*lngA*, E9034AΔ*cstH*, E9034AΔ*cstH*Δ*lngA*, and E9034AΔ*lngA*Δ*cstH*Δ*fliC*.

TLRs play crucial roles in recognizing the various antigenic CFs of *E. coli*, which activate signaling pathways that lead to the production of cytokines, such as IL-8. The findings described in this study suggest that CS21, CS3, and the ETEC flagellum are potential targets for vaccine development aimed at reducing diarrheal diseases caused by ETEC. Nevertheless, further research is necessary to fully characterize the immune response triggered by ETEC CFs. The detection of IL-8 will allow us to understand the immunological mechanisms involved in the immune response induced by ETEC in its host. A thorough characterization of the immune response elicited by different CFs will provide essential guidance for preventing and controlling diarrhea caused by ETEC.

## 5. Conclusions

Mutation of the *lngA* (CS21), *cstH* (CS3), and *fliC* (flagella) genes in the ETEC strain E9034A affected the ability of the bacterium to adhere to intestinal HT-29 and HuTu 80 cells. Mutations in the *fliC* gene directly impacted the ability of the bacterium to induce the release of the cytokine IL-8. The assembly of CS21, CS3, and flagellar structures in the bacterial outer membrane enhanced the adherence of ETEC to intestinal cells. Additionally, flagella played a role in inducing the host immune response; however, additional studies are needed.

On the other hand, elucidation of the genome of the E9034A ETEC strain using hybrid sequencing technology is essential for understanding the diverse factors that allow this bacterium to colonize humans, especially in the pediatric population. Advances in genetic characterization will facilitate future research into the virulence mechanism of ETEC, which could significantly impact pediatric health and contribute to the development of specific strategies to mitigate the effects.

## 6. Limitations

In this study, we investigated the functions of the LngA, CstH, and FliC proteins in ETEC through in vitro experiments. Importantly, further in vivo studies are needed to fully understand how the absence of these proteins affects bacterial adherence and how their expression enhances the ability of ETEC to colonize its host. Additionally, in vivo models will provide insights into how these proteins promote the release of pro- or anti-inflammatory cytokines. In summary, animal models will contribute more accurate information regarding the relationship between bacteria and the host immune system. These in vivo studies will be conducted in the next phase to deepen our understanding of the colonization processes and immune responses elicited by ETEC.

## Figures and Tables

**Table 1:** ETEC strain E9034A and its mutagenic variants.

ETEC Strain	Resistance	Serotype	LT	ST	CS21	CS3	CFA/I	CS1	CS8	Flagella	Origin
E9034A	--	O8:H9	+	+	+	+	+	+	+	+	Caribbean
E9034AΔ*lngA*	Km	O8:H9	+	+	−	+	+	+	+	+	Collection
E9034AΔ*fliC*	Cm	O8:H9	+	+	+	+	+	+	+	−	This study
E9034AΔ*cstH*	Cm	O8:H9	+	+	+	−	+	+	+	+	This study
E9034AΔ*lngA*Δ*fliC*	Km/Cm	O8:H9	+	+	−	+	+	+	+	−	This study
E9034AΔ*cstH*Δ*lngA*	Km/Cm	O8:H9	+	+	−	−	+	+	+	+	This study
E9034AΔ*cstH*Δ*fliC*	Km	O8:H9	+	+	+	−	+	+	+	−	This study
E9034AΔ*lngA*Δ*cstH*Δ*fliC*	Km/Cm	O8:H9	+	+	−	−	+	+	+	−	This study
Type strains used in this study											
BL21 (D3)	*E. coli* K-12 *fhuA*2 [lon] ompT gal (λ DE3) [dcm] Δ*hsdS* λ DE3 = λ sBamHIo ΔEcoRI-B int::(*lacI*::*Plac*UV5::T7 gene1) i21 Δnin5										

Enterotoxigenic *Escherichia coli* E9034A, Type IV pilus (CS21), fibrilla (CS3), Type IV pilus (CS1), Type IV pilus (CS8), Fimbria (CFA/I) [17]. Kanamycin (Km), chloramphenicol (Cm). Toxins: thermolabile (Lt), thermostable (St), positive (+) and negative (−).

**Table 2:** Primers used for amplification of the *lngA*, *cstH*, and *fliC* genes from the E9034A and mutant strains.

Gene	Primer	Sequence (5′–3′)	Size (bp)		References or Source
			Wild type	Mutant	
*cstH*	F	CTTAAGCTACATGCACAGGAGTAGC	710	1800	This study
*cstH*	R	GATACAGGAGCAGAATTACAAGCTTGACTATTT			
*lngA*	F	ATGAGCCTGCTGGAAGTTAGCATTGTTCTTGGCATTATCGGTACGATTGC	760	1800	22
*lngA*	R	TTAACGGCTACCTAAAGTAATTGAGTTTACCTGAGCAGTACAGGTACTTA			
*fliC*	F	ATGGCACAAGTCATTAATACCAAC	2010	1280	This study
*fliC*	R	TTAACCCTGCAGCAGAGACAGAA			
*lngA*	Rec R	GTGGTGGTGATGGTGATGGCCACGGCTACCTAAAGTAATTGAGTTTA	745		This study
*lngA*	Rec F	AGAAGGAGATATAACTATGCTGTATAACCGG			
*cstH*	Rec F	AGAAGGAGATATAACTATGTTAAAAATAAAATACTTATTAATAGGTCT TTCACTGTCAGCTAT	510		This study
*cstH*	Rec R	AGAAGGAGATATAACTATGTTAAAAATAAAATACTTATTAATAGGTCT TTCACTGTCAGCTAT			
*fliC*	Rec F	AGAAGGAGATATAACTATGGCACAAGTCATTAATACCAACAGCCTCTCG	745		This study
*fliC*	Rec R	GTGGTGGTGATGGTGATGGCCACCCTGCAGCAGAGACAGAA			

Forward (F), Reverse (R), Recombinant (Rec).

## Data Availability

The complete genome of the ETEC strain E9034A has been deposited in NCBI, including its chromosome (accession number: CP171231), with a length of 4,801,745 bp, and three plasmids (accession numbers: CP171232, CP171233, and CP171234), with sizes of 76,272 bp, 57,583 bp, and 3237 bp, respectively. This genomic information is associated with BioProject PRJNA1171086. Currently, these data are not available to the public because they will be used for publication by the research group; they will be released to the public after one year.

## Supplementary Materials

The following supporting information can be downloaded at: www.mdpi.com/article/10.3390/microorganisms13020374/s1, File S1: The adherence data of ETEC strains on HT-29 and HuTu-80 cells.
