# Supplementary material for: Genome Sequencing and Assembly of Enterotoxigenic Escherichia coli E9034A: Role of LngA, CstH, and FliC in Intestinal Cell Colonization and the Release of the Proinflammatory Cytokine IL-8
Source: Microorganisms. 2025 Feb 8;13(2):374. doi: 10.3390/microorganisms13020374 (PMC11858209; doi:10.3390/microorganisms13020374)
Supplement: Supplementary file 1 [file microorganisms-13-00374-s001.zip › microorganisms-3469292-supplementary.pdf]

Supplementary Material File S1. The adherence data of ETEC strains on HT-29 and HuTu-80 cells.

**Adherence assays of E9034A and mutant strains in HT29 cells and without cells**				
Strains	A: HT29 cells (CFU/mL)	B: Without HT29 cells (CFU/mL)	CFU/mL (A-B)	CFU 1X10^6/mL
E9034A	19200000	5187	19194813	19.19
E9034AΔ*lngA*	13600000	3962	13596038	13.59
E9034AΔ*cstH*	9700000	3875	9696125	9.6
E9034AΔ*fliC*	14800000	3875	14796125	14.79
E9034AΔ*lngA*Δ*fliC*	11300000	6425	11293575	11.29
E9034AΔ*cstH*Δ*lngA*	10600000	6487	10593513	10.59
E9034AΔ*cstH*Δ*fliC*	9100000	4437	9095563	9.09
E9034AΔ*cstH*Δ*fliC*Δ*lngA*	4700000	3312	4696688	4.69

This table represents the values obtained from the adhesion tests in HT-29 cells, those obtained without cells, and the subtraction of both values.

**Adherence assays of E9034A and mutant strains in HuTu 80 cells and without cells**				
Strains	A: HuTu 80 cells (CFU/mL)	B: Without HuTu 80 cells (CFU/mL)	CFU/mL (A-B)	CFU 1X10^6/mL
E9034A	26830000	1110	26828890	26.82889
E9034AΔ*lngA*	7160000	5440	7154560	7.15456
E9034AΔ*cstH*	17160000	330	17159670	17.15967
E9034AΔ*fliC*	11000000	330	10999670	10.99967
E9034AΔ*lngA*Δ*fliC*	5830000	4800	5825200	5.8252
E9034AΔ*cstH*Δ*lngA*	14160000	1110	14158890	14.15889
E9034AΔ*cstH*Δ*fliC*	4660000	1110	4658890	4.65889
E9034AΔ*cstH*Δ*fliC*Δ*lngA*	2500000	1000	2499000	2.499

This table represents the values obtained from the adhesion tests in HuTu 80 cells, those obtained without cells, and the subtraction of both values.
